# Microstructure Evolution and Mechanical Properties of Underwater Dry and Local Dry Cavity Welded Joints of 690 MPa Grade High Strength Steel

**DOI:** 10.3390/ma11010167

**Published:** 2018-01-22

**Authors:** Yonghua Shi, Kun Sun, Shuwan Cui, Min Zeng, Jianglong Yi, Xiaoqin Shen, Yaoyong Yi

**Affiliations:** 1 School of Mechanical and Automotive Engineering, South China University of Technology, Guangzhou 510640, China; 13597066615@163.com (S.C.); memzeng@scut.edu.cn (M.Z.); xiaoqinshen@163.com (X.S.); 2 Guangdong Provincial Engineering Research Center for Special Welding Technology and Equipment, South China University of Technology, Guangzhou 510640, China; 3 Guangdong Welding Institute (China-Ukraine E. O. Paton Institute of Welding), Guangzhou 510650, China; yijl@gwi.gd.cn (J.Y.); yiyy@gwi.gd.cn (Y.Y.)

**Keywords:** underwater welding, microstructure, mechanical properties, high strength steel

## Abstract

Q690E high strength low alloy (HSLA) steel plays an important role in offshore structures. In addition, underwater local cavity welding (ULCW) technique was widely used to repair important offshore constructions. However, the high cooling rate of ULCW joints results in bad welding quality compared with underwater dry welding (UDW) joints. Q690E high strength low alloy steels were welded by multi-pass UDW and ULCW techniques, to study the microstructural evolution and mechanical properties of underwater welded joints. The microstructure and fracture morphology of welded joints were observed by scanning electron microscope and optical microscope. The elemental distribution in the microstructure was determined with an Electron Probe Microanalyzer. The results indicated that the microstructure of both two welded joints was similar. However, martensite and martensite-austenite components were significantly different with different underwater welding methods such that the micro-hardness of the HAZ and FZ in the ULCW specimen was higher than that of the corresponding regions in UDW joint. The yield strength and ultimate tensile strength of the ULCW specimen are 109 MPa lower and 77 MPa lower, respectively, than those of the UDW joint. The impact toughness of the UDW joint was superior to those of the ULCW joint.

## 1. Introduction

Underwater welding has become the main method employed for the repair and maintenance of offshore oil and gas pipelines, mining platforms, watercraft, seashore components, piers as well as harbor devices and systems. Underwater arc welding technology is used for underwater wet welding, underwater dry welding (UDW), and underwater local cavity welding (ULCW). During underwater wet welding, the welding process is directly exposed to the water environment. However, UDW is performed in a chamber that is sealed around the structure to be welded. The chamber is filled with gas at atmospheric pressure or a pressure corresponding to the water depth. During ULCW, a small region is evacuated to create a dry spot, which is used to protect the stable welding process. Therefore, this method is cheaper and more convenient than UDW and raises higher quality welded joints than underwater wet welding. The cooling rate employed after welding by ULCW is different from that used in wet underwater welding and in-air welding [1]. Similarly, owing to a difference in welding environments, the quality of underwater local dry cavity welds differs significantly from that of dry welds. 

The offshore environment is multi-variate with waves, wind, and currents all contributing to the forces experienced by offshore systems [2]. Therefore, high strength, excellent ductility, and high toughness at −40 °C represent critical properties for large offshore structures operating in this complex environment. High strength low alloy (HSLA) steel plays an important role in offshore structures, owing to their excellent ductility, low-temperature toughness, and weldability. With the rapid development of the marine economy, HSLA steels have been widely applied in these structures.

In the manufacturing of offshore structures, underwater welding constitutes an essential manufacturing technique for joining and repairing important constructions. High-quality underwater welding rises improved quality of ocean constructions, and mitigates the complexity of manufacturing large structures. The joints microstructure and properties are determined by the chemical composition of the material, the cooling rate employed after welding, and the welding methods applied. Bayley et al. [3] reported that traditional arc welding procedures can produce a soft heat affected zone (HAZ), owing to the relatively high heat input and slow cooling rates of the weldment. The soft HAZ generally leads to low strength of the weldment. During multi-pass welding, regions of poor impact toughness within the HAZ have an adverse effect on HSLA steels. One of these regions is referred to as the intercritically reheated coarse grained HAZ or intercritical zone [4]. Lambert et al. [5] determined the effect of martensite-austenite constituents (M-A) within HSLA steel welds on the toughness properties and found that the bainitic microstructure and M-A constituents strongly affect the impact properties of simulated HAZ. In addition, compared with retained austenite, high-carbon martensite has a significantly more deleterious effect on these properties. Byno et al. [6] reported that, during the welding of HSLA, the heat input increased and the cooling rate decreased. This resulted in increased cellular dendritic cell spacing, decreased acicular ferrite content, and an increase in the size of acicular ferrite laths. During the welding of HSLA, the occurrence of M increases with increasing cooling rate, and has a negative effect on the mechanical properties of welds. Di et al. [1] simulated the local dry underwater welding process of E550 steel. They concluded that rapid cooling rate can improve the impact toughness and tensile strength of weld metal in local dry underwater welding. Fydrych et al. [7] studied the weldability of high strength steel in underwater environment. They concluded that joints of steel S355J2G3 and S500M created underwater in conditions of restraint showed a strong tendency to form cold cracks in the weld. Cold cracks were found in all Tekken test joints of steel S500M created by wet welding, whereas for test joints made in air cracks were found in only one of the two joints.

The difference in the cooling rate of local dry cavity welds and dry welds results in differences in the welding quality. Zhai et al. [8] simulated the local dry underwater tungsten inert gas welding with a flux-cored wire and analyzed high-speed photographs of the metal transfer process. They concluded that the metal transfer mode varies from the no-contact mode/slag column mode to the continuous liquid transfer mode, with an increase in the wire-electrode gap. The appearance of the weld pool varies greatly with the change in the metal transfer mode. Guo et al. [9] studied the effects of arc voltage on the stability of the underwater wet flux-cored arc welding (FCAW-S 114) process. They concluded that the mode of metal transfer and the protective bubble effect, which vary with voltage, affect the stability of the welding process. However, detailed studies of the difference in the joint microstructures and mechanical properties of ULCW and UDW joints are lacking. In the current work, workpieces of Q690E HSLA steel are welded by ULCW and UDW, respectively. The characteristics and performance of these joints are evaluated by comparing the corresponding microstructures, tensile properties as well as the micro-hardness and Charpy impact toughness values. The results will be useful in determining a welding procedure for the underwater welding of Q690E steel.

## 2. Experimental Materials and Procedure

### 2.1. Experimental Materials

**Figure 1:**
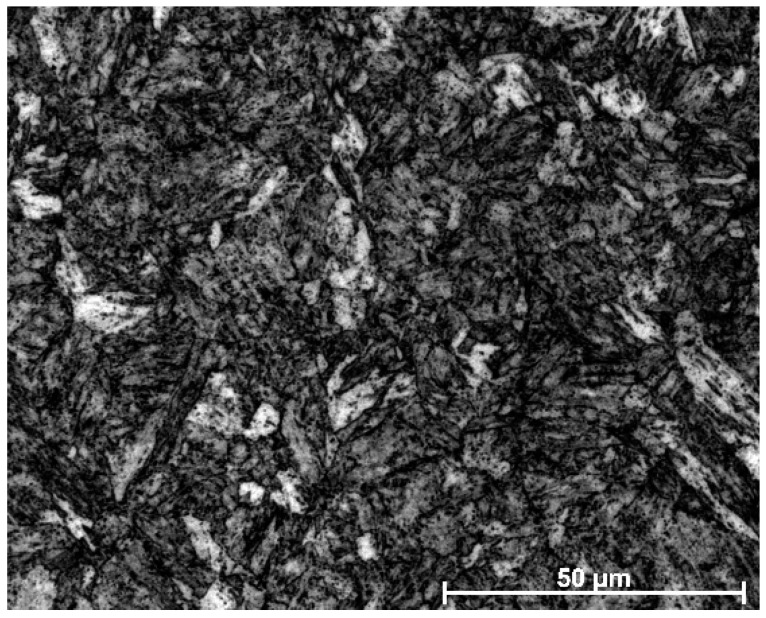
Microstructure of Q690E HSLA (High strength low alloy) steel.

The base material (BM) used in this study, which was supplied by Sha Steel, was in the form of 10 mm thick Q690E HSLA steel with a tensile strength of 690 MPa (see Figure 1 for microstructure and Table 1 for mechanical properties). Table 2 present the chemical compositions (by wt. %) of the BM and the filler wire. The BM was produced through a Quenching and Tempering (QT) process, where the steel was heated to 930 °C and water quenched, then reheated to 630 °C, and subsequently air-cooled to room temperature. The microstructure of the BM consisted mainly of tempered martensite (TM) and a small amount of bainite (B).

### 2.2. Welding Process

**Figure 2:**
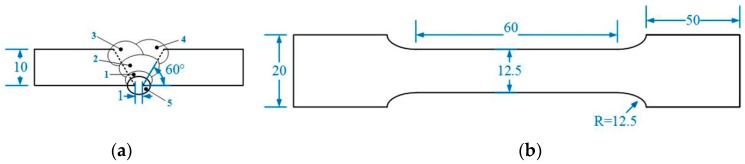
Welding pass sequence and tensile specimens: (**a**) Welding pass sequence; (**b**) Dimensions of the tensile specimens (the thickness of tensile specimens is 8 mm; all dimensions are in millimeter).

Using Mn3Ni1CrMo filler wires, 300 × 100 × 10 mm^3^ workpieces of Q690E steel were welded by gas metal arc welding (GMAW); see Table 2 for the chemical compositions (by wt. %) of the BM and the filler wire. Experiments of underwater dry welding and local cavity welding were conducted in a hyperbaric chamber, where an automatic underwater welding system was located. The experimental system consisted of a high-pressure chamber, welding power source, three-dimensional motion platform, and auxiliary equipment. Prior to welding, compressed air was let into the chamber to simulate the pressure associated with the water depth. Typically, a pressure of 0.1 MPa is used to simulate the pressure corresponding to a water depth of 10 m. The butt joints were fabricated through multi-pass welding (see Figure 2). During UDW, the hyperbaric chamber contains no water. Each welding pass was cooled to 100 °C by natural air prior to the subsequent pass. Welding experiments were accomplished at water depths of 0.3 m. This differed from the other welding process where, prior to ULCW, water was poured into the chamber until the water surface was 0.3 m higher than the workpiece surface. Therefore, during ULCW, each welding pass was followed by water cooling to room temperature. The diameter of welding wire was 1.2 mm and the chemical composition of shielding gas was 80% Ar_2_ + 20% CO_2_. During underwater local cavity welding, the value of gas flow was 50–55 L/min, while this value was 25–30 L/min during underwater dry welding. The shape of cavity is cylindrical container with the diameter of 30mm and the height of cavity is 20 mm. The underwater welding parameters associated with various conditions are listed in Table 3. For each welding experiment, the required heat input can be calculated from:

(1) q=ηUI/v

where *q*, *I*, *U*, *v*, and *η* are the heat input (J), welding current (A), arc voltage (V), welding speed (mm·s^−1^), and arc efficiency, respectively. For gas metal arc welding, η = 0.85 [10].

### 2.3. Mechanical Tests

In accordance with the ASTM E384-17 standard [11], the Vickers micro-hardness mapping profiles across the welded joints were measured using a load of 300 g. The holding time for measurements was 15 s. Three measurements were performed at each site. 

Tensile test specimens (see Figure 2b for size and geometry of the specimens) of the BM and the welded joints were prepared in accordance with ASTM E8M-16a [12]. The specimens were fabricated by electric discharge machining (EDM) of the weld metals perpendicular to the welding direction and subsequently tested at room temperature. Each specimen consisted of the weld metal, heat affected zone (HAZ) on both sides, and the BM. 

Specimens (55.0 × 10.0 × 5.0 mm^3^) for Charpy V notch (CVN) impact toughness evaluation were prepared in accordance with ISO 148-1 [13]. The impact tests were performed at −40 °C on a Charpy impact testing machine (MTS ZBC2303-3, Shenzhen, China). Nital solution was used to increase the visibility of the outline of the fusion line and the HAZ. Furthermore, the impact toughness of the weld metal and HAZ was determined from CVN positions located in each region. Experimental uncertainties were reduced by performing three replicates of each impact test. After the tensile and Charpy impact tests, the fracture surfaces of the tested specimens were all examined by scanning electron microscopy (SEM, LEO 1530 VP, Oberkochen, Germany).

## 3. Results and Discussion

### 3.1. Effects of Welding Process on Microstructures

**Figure 3:**
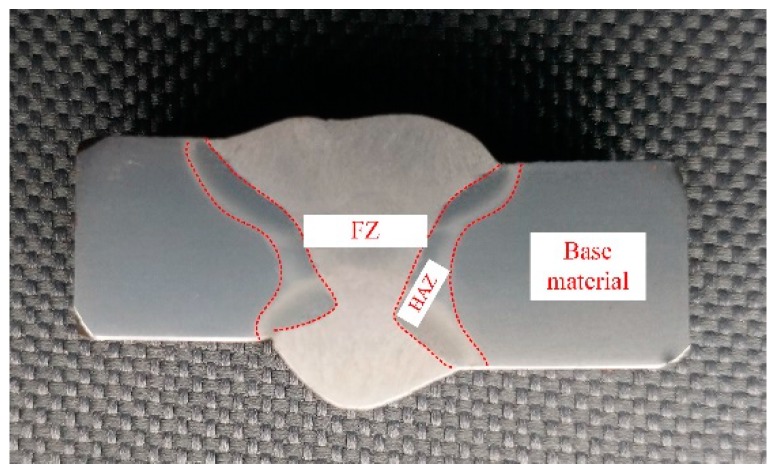
Cross-section profile of the ULCW (underwater local cavity welding) joint of Q690E steel.

**Figure 4:**
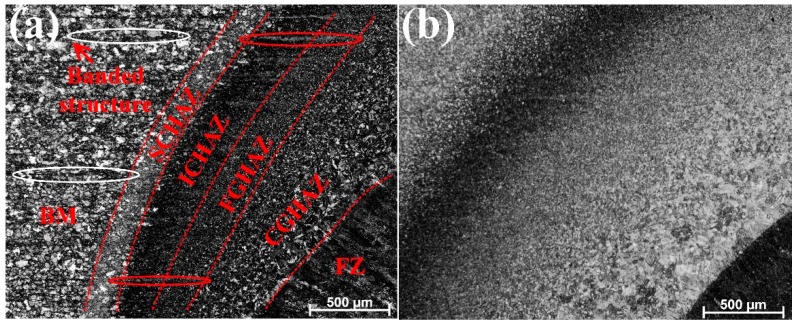
Microstructures of different zones within the welded joints: (**a**) ULCW joint; (**b**) UDW (underwater dry welding) joint.

The macrograph of a ULCW joint of Q690E steel (Figure 3) reveals three distinct regions namely, the fusion zone (FZ), HAZ, and BM. During the welding process, the peak temperature varies with position in the HAZ and, hence, the sub-zones of the HAZ are characterized by different microstructures. Based on the microstructure, the HAZ can be further divided into four different sub-zones, i.e., the: coarse-grained HAZ (CGHAZ), fine-grained HAZ (FGHAZ), intercritical HAZ (ICHAZ), and sub-critical HAZ (SCHAZ) adjacent to the BM, as shown in Figure 4.

**Figure 5:**
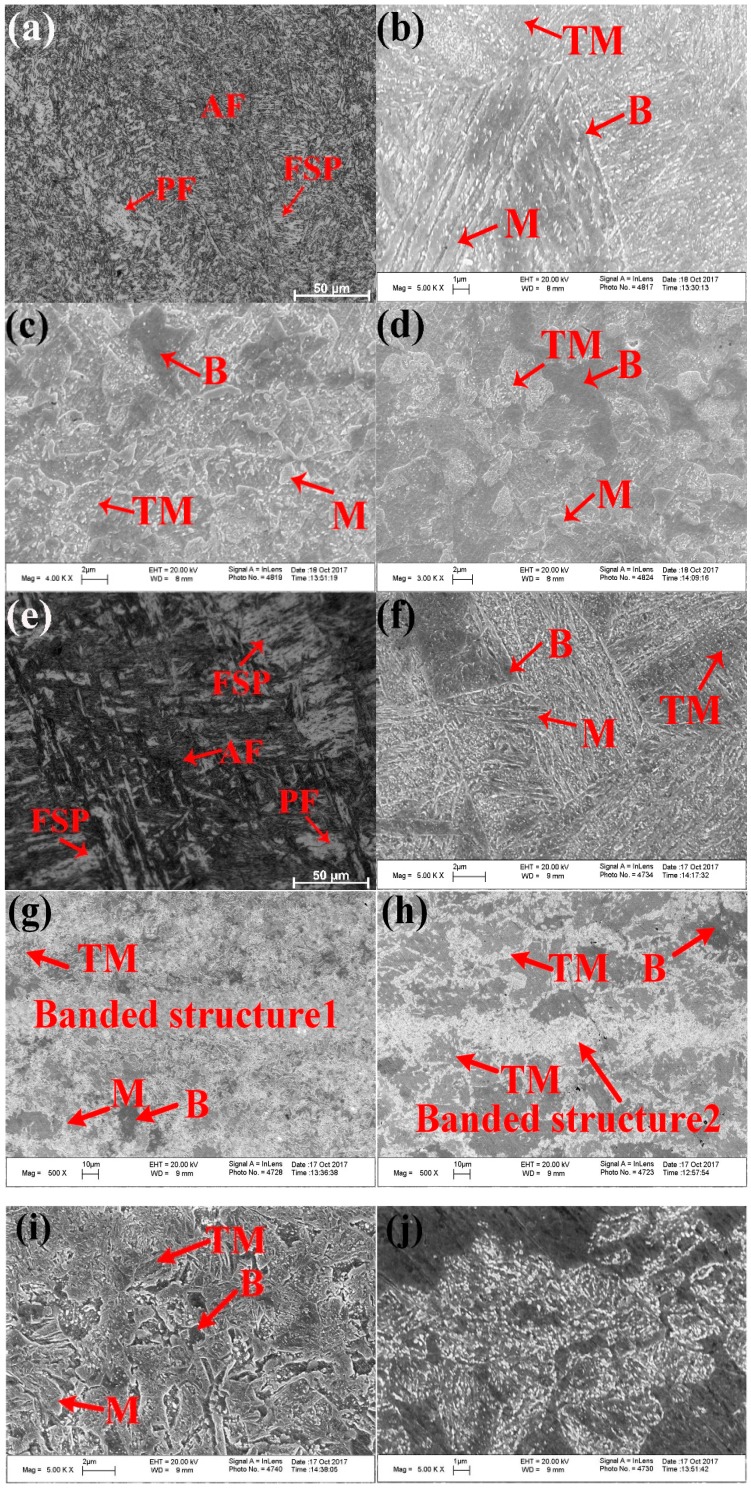
OM (optical microscopy) and SEM (scanning electron microscopy) micrographs showing the microstructures in different sub-zones of the welded joints: (**a**) FZ-UDW joint; (**b**) CGHAZ-UDW joint; (**c**) ICHAZ-UDW joint; (**d**) ICHAZ-UDW joint; (**e**) FZ-ULCW joint; (**f**) CGHAZ-ULCW joint; (**g**) ICHAZ-ULCW joint; (**h**) SCHAZ-ULCW joint; (**i**) Banded structure1; (**j**) Banded structure2.

The microstructural evolution within each sub-zone of the welded joints is controlled mainly by the peak temperature and the cooling rate. The microstructures comprising each of the sub-zones (SCHAZ, ICHAZ, FGHAZ, CGHAZ, and FZ) of the UDW joint and the ULCW joint are examined by optical microscopy (OM) and SEM; the corresponding micrographs are shown in Figure 4 and Figure 5, respectively. Above the Ac_3_ temperature, the CGHAZ and FGHAZ experience high peak temperatures, resulting in re-austenization of the original grains. The CGHAZ experiences the highest peak temperature and remains above the Ac_3_ temperature for the longest period, leading to an increase in the grain size. Therefore, the CGHAZ has the largest grain size in both the UDW and ULCW joints (see Figure 4). This grain size decreases, however, with increasing distance from the fusion line. 

The FZ is melted during the heating process, and subsequently solidifies during the cooling process. As shown in Figure 5a or Figure 5e, the microstructures of FZ within the UDW joint and ULCW joint consist mainly of three types of ferrite: acicular ferrite (AF), proeutectoid ferrite (PF), and ferrite side plate (FSP). Compared with those of the UDW joint, the No. 4 (see Figure 5e for microstructure) and 5 passes of FZ within the ULCW joint yield more PF and FSP. This results from the fact that the cooling rate, due to the water quenching effect, of the ULCW joint is higher than that of the UDW joint [1]. Moreover, the No. 5 pass of FZ is performed without a subsequent welding cycle and duration of the No. 4 pass of FZ are shorter, because of water quench following the No. 4 and 5 pass and without the experience of subsequent reheating from neighboring pass. The ferrite distribution associated with other passes of FZ within the ULCW joint is similar to the ferrite distribution of the UDW joint. We identify the different phases by SEM examination of the microstructures composing the CGHAZ in both joints (see Figure 5b or Figure 5f). After ULCW, the coarse-grained austenite transforms completely to martensite (M) and a small amount of B, owing to water quenching (Figure 5f). Some of this M then transforms to TM due to tempering after the welding process. After UDW, the austenite transforms to M and some B. This M can self-temper during the subsequent cooling process, owing to the relatively low cooling rate and tempering after the welding process. Therefore, CGHAZ of the UDW joint contains more TM than CGHAZ of the ULCW joint, but both zones consist of a mixture of equiaxed M, B, and auto-TM. The peak temperature of FGHAZ is slightly higher than the Ac_3_ temperature. Although the material in this region also undergoes complete re-austenization, short periods of time at these temperatures limit the austenite grain growth of the FGHAZ. This limited growth raises a fine austenite grain size within the FGHAZ. Moreover, the microstructures in the FGHAZ are similar to those of the CGHAZ in the UDW joint and the ULCW joint.

At the same time, the ICHAZ is exposed to temperatures lying between the Ac_1_ and Ac_3_ temperatures, and the grains in this region are partially transformed into austenite. During the rapid cooling process of the ULCW joint, the austenite transforms to M and B, and the BM microstructure is retained in the other regions (Figure 5g). The cooling rate of the ULCW joint is higher than that of the UDW joint and, hence, more M form in the ICHAZ of the ULCW joint. In the ICHAZ of the UDW joint, the un-transformed base material is over-tempered by the welding thermal cycle. Some of the austenite is partially transformed to M and B, and the BM microstructure is retained in other regions (Figure 5c). Figure 5d,h show the microstructures composing the SCHAZ of the UDW joint and the ULCW joint, respectively. The peak temperatures of the SCHAZ are lower than the Ac_1_ temperature and, therefore this region undergoes re-tempering [14]. Similar to that of the BM, the microstructure of SCHAZ consists of a mixture of TM and B.

As shown in Figure 4a, two types of banded structures form in the FGHAZ, ICHAZ, SCHAZ and BM of the ULCW joint. Banded structure1 was circled by red ellipse and banded structure2 was circled by white ellipse. Etching the ULCW joint with Lepera etchant revealed that M banded structures are the main banded structures formed in the CGHAZ and ICHAZ. We examine banded structure1 (Figure 5i) and banded structure2 (Figure 5j) by SEM. The SEM micrographs revealed that banded structure1 is a mixed structure composed mainly of M, TM, and B, and banded structure2 consists of TM. Chemical inhomogeneity and hot rolling yield a banded structure in HSLA, which (owing to strain anisotropy) is detrimental to the mechanical properties of steel [15]. During the ULCW process, the ULCW joint is subjected to multiple rapid heating and cooling (water quenching) steps, which reduced the chemical homogeneity of the material. During the UDW process, preheating, maintaining the inter-pass temperature at 100 °C, and air cooling lead to a decrease in the cooling rate during UDW. This yield increased time for diffusion of the alloying elements thereby mitigating chemical inhomogeneity. Further, the less banded structure in the UDW joint could be due to inevitable variations of chemical inhomogeneity between different pieces of the base plate, which is a common feature of lab-made materials.

**Figure 6:**
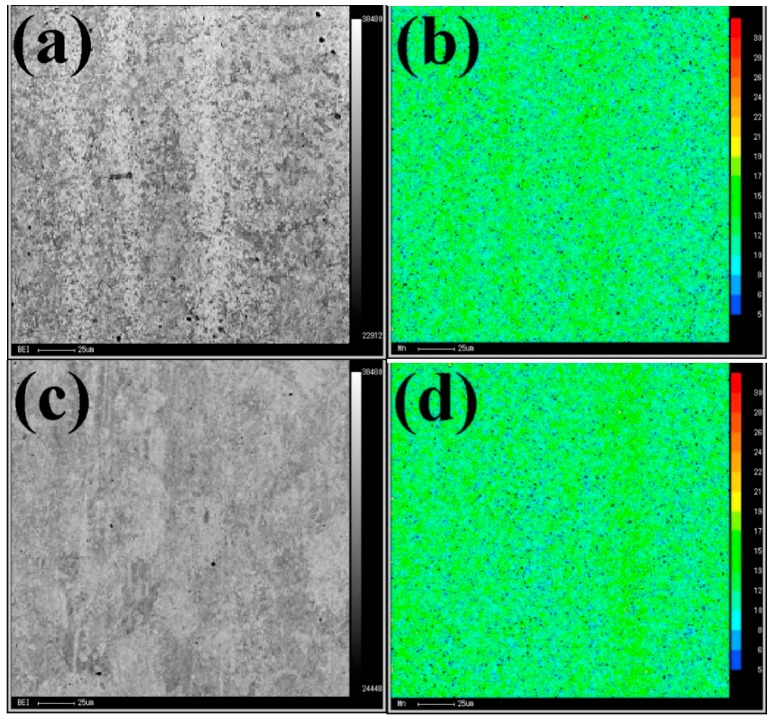
EPMA (Electron Probe Microanalyzer) elemental distribution maps in banded structures: (**a**) backscattered electron image (BEI) of banded structure1; (**b**) distribution of manganese within banded structure1; (**c**) BEI of banded structure2; and (**d**) distribution of manganese within banded structure2.

The elemental distribution of Mn in the banded structures (see Figure 6) was determined with an Electron Probe Microanalyzer (EPMA). In Figure 6b or Figure 6d, blue and green represent low content and medium content, respectively, while yellow and red correspond to high content. Mn is inhomogeneously distributed in both banded structures. As Figure 6b shows, Mn-segregated bands parallel to the rolling direction occur in the FGHAZ and ICHAZ of the ULCW joint. The Mn-poor bands (level value: 5–12; unit: X-ray counts) and Mn-rich bands (level value: 12–21) are composed of B and M, respectively. The Mn-poor bands (level value: 5–12) are mainly composed of TM, and Mn-rich bands (level value: 12–21) within the SCHAZ of the joint are mainly composed of B (Figure 6d). The occurrence of banded structures is attributed to the inhomogeneous distribution of Mn in welded joints.

**Figure 7:**
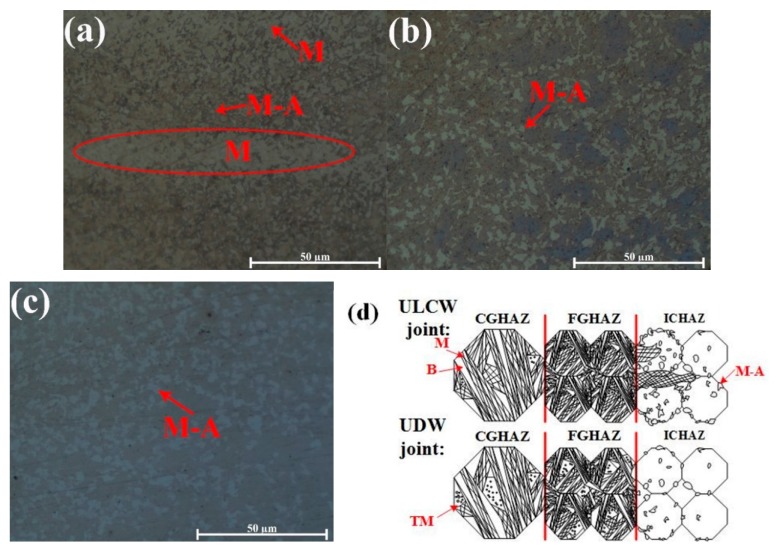
M-A constituents revealed by Lepera etchant: (**a**) the starting region of the ICHAZ-ULCW joint; (**b**) the tail region of the ICHAZ-ULCW joint; (**c**) ICHAZ-UDW joint; (**d**) the distribution of M and M-A components within HAZs.

The distribution and fraction of martensite–austenite islands (M–A) within ICHAZ (see Figure 7a–c) is revealed through etching with Lepera etchant. The distribution of M and M-A components within the HAZ of the ULCW joint and the UDW joint are schematically illustrated in Figure 7d. Compared with the UDW joint, the CGHAZ of the ULCW joint has less TMs and Bs, but more Ms, owing to water quenching. Different trends are observed for the FGHAZ of the ULCW joint. Compared with that occurring in the ICHAZ of the UDW joint, (i) more M-banded structures form in the starting region of the ICHAZ of the ULCW joint, and (ii) the formation of M-A components is more likely along grain boundaries within the ICHAZ of the ULCW joint. These components also form along the grain boundaries at another edge of the ICHAZ of the ULCW joint. In contrast, a uniform distribution of M-A components is formed at the starting region of the ICHAZ of the UDW joint. The amount of M-A components decreases with increasing distance to the FGHAZ within both joints. These components are almost absent from another edge of the ICHAZ of the UDW joint.

### 3.2. Micro-Hardness

**Figure 8:**
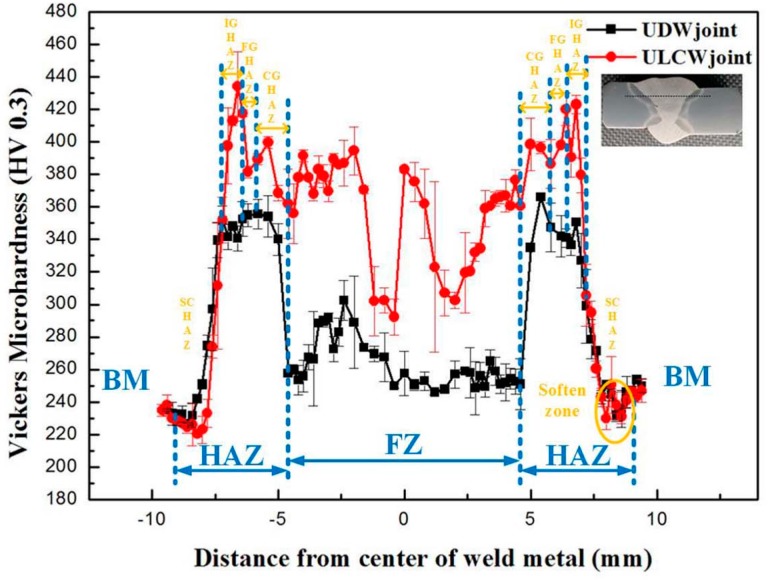
Micro-hardness measurements on the welded joints.

The variation in micro-hardness across the weld zone is shown in Figure 8. The FZ, HAZ, and BM can be clearly distinguished based on this variation. The micro-hardness of the material is influenced mainly by the microstructure and grain size. Generally, the different phases in steel can be arranged in descending order of micro-hardness magnitude, as follows: martensite > bainite > pearlite > ferrite [16].

The FZ, HAZ, and BM zones of both specimens may be arranged in descending order of micro-hardness magnitude, as follows: HAZ > FZ > BM (average micro-hardness of BM: ~256 HV_0.3_). The hardness of the HAZ may be attributed to the fact that both welds are subjected to a non-uniform welding thermal cycle, which rises an inhomogeneous microstructure (see Figure 4 and Figure 5). These hardness values improve with the formation of M and B within HAZ. As shown in Figure 5a or Figure 5e, the microstructure of each FZ consists of a mixture of PF, FSP, and fine-grained AF. In addition, the Mn concentration (1.6%, see Table 2) and, hence, the micro-hardness of the FZs are higher than those of the BM [17].

The micro-hardness values of the FZ (average: ~370 HV_0.3_) and HAZ in the ULCW joint are higher than those of the corresponding regions of the UDW joint (FZ average micro-hardness: ~270 HV_0.3_, see Figure 8). However, similar micro-hardness values are obtained for the BM regions of the joints. Water quenching following the welding process of the ULCW joint results in an increase in the dislocation density of the microstructure. This dislocation density, which is higher than those of the FZ of the UDW joint, yield improved micro-hardness of the FZ. As Figure 5 and Figure 6 show, the microstructure of each sub-zone (CGHAZ, FGHAZ, and IGHAZ) of the ULCW joint contains significant amounts of M and B. The rapid cooling rate of the ULCW joint may result in an increase in the dislocation density of the microstructure composing these zones and, in turn, improved micro-hardness of the HAZs in this joint.

As Figure 8 shows, the peak micro-hardness (~430 HV_0.3_) of the HAZ-ULCW joint occurs for the ICHAZ, which consists primarily of M, M-A, B, and TM (see Figure 5g). Several carbides and a significant amount of Ms and M-As (Figure 5i) with high hardness precipitated in this zone, due to the relatively high cooling rate and the tempering effect associated with the subsequent welding passes. In contrast, the peak micro-hardness of the HAZ-UDW joint (~360 HV_0.3_) occurs in the CGHAZ that consists mainly of M with a mixture of B and TM (Figure 5b), which increases the micro-hardness. The micro-hardness of both joints decreases rapidly from the CGHAZ to the SCHAZ, as shown in Figure 8. Moreover, the peak temperature of the sub-zones decreases (from Ac_1_ to 630 °C) from the CGHAZ to the SCHAZ. Therefore, the homogeneity of the microstructure increases and the M and M-A regions gradually disappear. The micro-hardness of the softened zone within the SCHAZ (~220 HV_0.3_) is lower than that of the BM. This is attributed to the fact that the SCHAZ undergoes high-temperature (although lower than the Ac_1_ temperature) re-tempering during the welding process. The re-tempering process may have resulted in a reduction in the dislocation density and softening of the microstructure comprising this zone [18].

### 3.3. Uniaxial Tensile Strength

**Figure 9:**
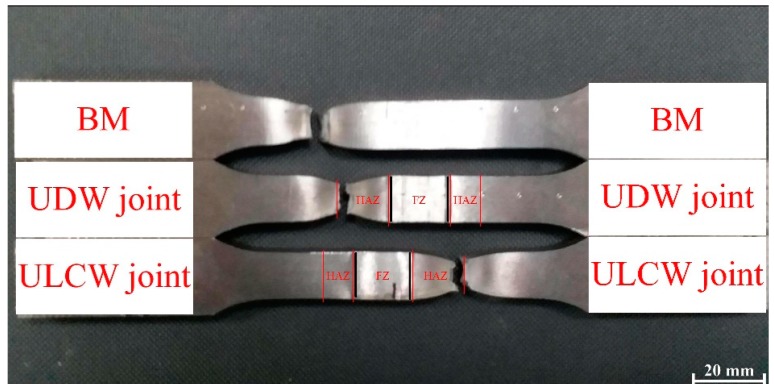
Fracture locations for the tensile test specimens.

The tensile specimens that underwent tensile testing at room temperature are shown in Figure 9. The tensile weld specimens all fail in the soft HAZ, but exhibit differing strength properties.

**Figure 10:**
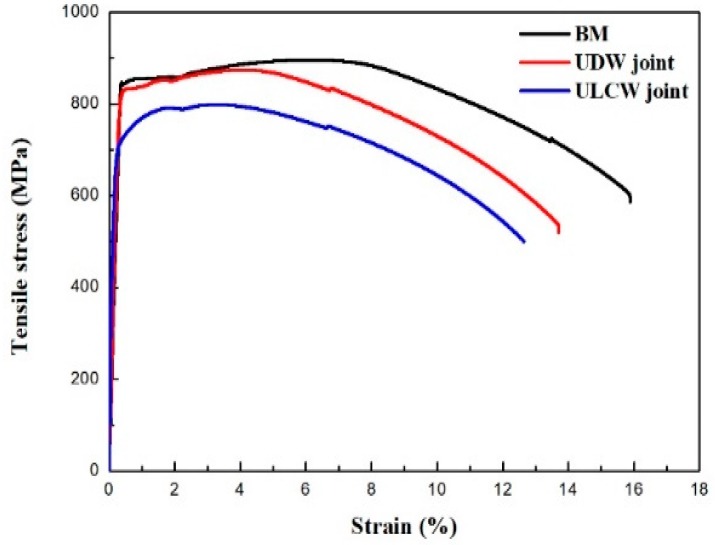
Tensile stress–strain curves of the base metal and welded joints.

The representative tensile stress-strain plots of the BM, UDW, and ULCW joints of Q690E steel are shown in Figure 10. The yield strength (YS) and ultimate tensile strength (UTS) of the joints and BM are summarized in Table 4. The welded specimens (especially the FZ and HAZ) are inhomogeneous and, hence, the values recorded for the YS and the UTS are non-representative of the sub-zones of the samples [18]. The BM specimen presents the best tensile properties. However, the tensile properties of the ULCW joint sample are inferior to those of the Q690E BM. The YS and UTS of the BM (843 MPa and 896 MPa, respectively) are higher than those (YS: 831 MPa, UTS: 876 MPa) of the UDW joint. However, the YS: 722 MPa and UTS: 799 MPa of the ULCW joint are ~120 MPa lower and 97 MPa lower, respectively, than those of the BM.

The micro-hardness is correlated with the yield strength and tensile strength, and an increase in the hardness results in increasing strength [19]. A comparison of the micro-hardness distributions (see Figure 8 and Figure 9) reveals that the tensile specimens all fail in the soft HAZ. Failure of all the tensile specimens in the soft HAZs indicates that these are the weakest region of the joints. This is attributed to the fact that the (i) high micro-hardness of regions in the FZ and HAZ (except for the SCHAZ) may result in improved yield strength and tensile strength, whereas the (ii) low micro-hardness of the softened zone may lead to a decrease in the welded-joint strength [20]. The harder FZ and the harder regions of the HAZ (compared with the softened zone) can act as a strong constraint on plastic deformation. Therefore, most of the tensile plastic deformation accumulates in the soft HAZ zones. However, under a uniaxial tensile stress, the BM specimen undergoes uniform deformation and, owing to the uniform microstructure, necking and fracture occur when an external load is applied [16]. The coarse necklacing M-A components occurring preferentially along the grain boundaries in the ICHAZ of the ULCW joint (Figure 7), have a negative effect on the strength. Therefore, the ULCW joint sample exhibits lower yield strength and tensile strength than the UDW joint.

**Figure 11:**
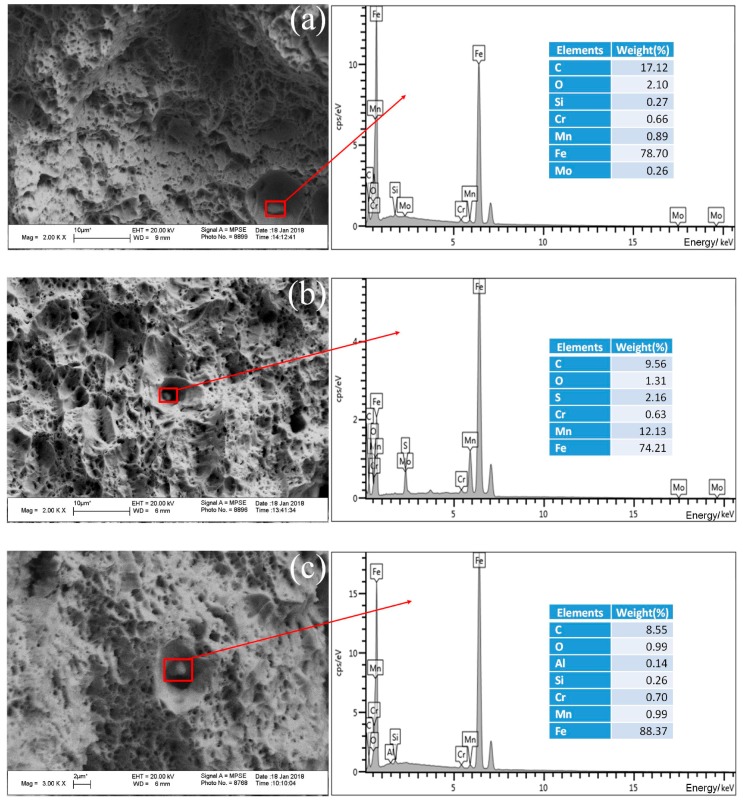
SEM micrographs showing the fracture surface morphology for the tensile test specimens, and the results of EDS (energy dispersive spectroscopy) analysis: (**a**) BM; (**b**) UDW joint; (**c**) ULCW joint.

The fracture mechanism of tensile test samples of the BM, UDW joint, and ULCW joint was identified by examining optical and scanning electron micrographs (Figure 11) of the surfaces of the failed specimens. The fracture surfaces are characterized by dimples of various sizes and depthsand a few small cleavage facets. This indicates that fracture of the BM sample is governed primarily by the quasi-cleavage fracture mechanism. Figure 11b,c show representative fractographs of the central fracture region of the UDW joint and the ULCW joint samples, respectively. The tensile fracture surface of both samples consists of cleavage facets and dimples of various sizes and depths. This indicates that fracture of both types of joints is governed by the quasi-cleavage fracture mechanism, although the ULCW joint has a lower YS and UTS than the UDW joint. In addition, many spherically shaped inclusion particles are present in the large voids of both types of joints.

As determined by energy dispersive spectroscopy (EDS), the inclusion particles within (i) BM contain Fe, Cr, Mn, Mo, O, Si, and C (see Figure 11a–c); (ii) the UDW joint contain Fe, Cr, Mn, S, O and C; and (iii) the ULCW joint are rich in Fe, Cr, Mn, Al, Si, O and C.

### 3.4. Charpy Impact Toughness

**Figure 12:**
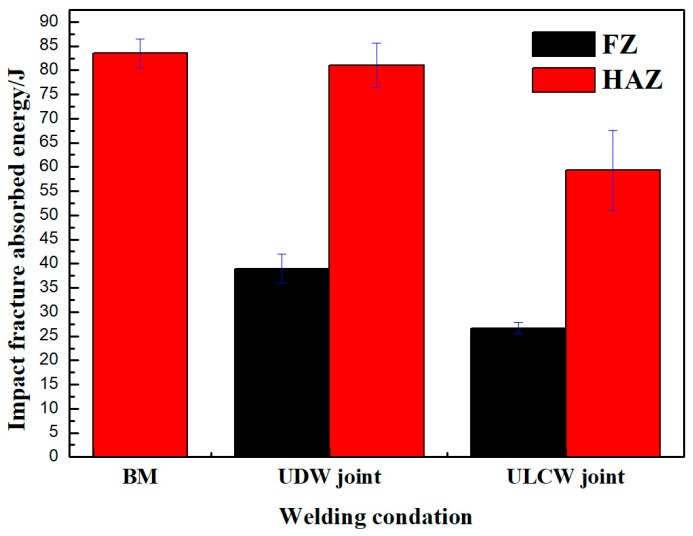
Absorbed energy results for the base material, weld wire, FZ (fusion zone) and HAZ (heat affected zone) impact specimens in UDW joint and ULCW joint at −40 °C.

Sub-sized (55.0 × 10.0 × 5.0 mm^3^) specimens are subjected to Charpy impact testing at a temperature of −40 °C. The original absorbed impact energy of the BM, UDW joint, and ULCW specimens with notches opened at the weld center line and in the HAZ region is shown in Figure 12 and Table 5.

The rapid cooling rate of the ULCW joint process results in increased generation of M in the FZ, CGHAZ, FGHAZ, and IGHAZ and, consequently, a decrease in the toughness of the joint. The results in Figure 12 and Table 5 show that: the absorbed energy values of the ULCW specimens with notches opened in the HAZ is lower than BM tested at −40 °C. The absorbed energy of the HAZ (59.3 J) is considerably lower than those of the HAZ of the UDW joint and the BM, suggesting that the HAZ of the ULCW specimen has lower toughness than these materials. Furthermore, the volume fraction of coarse necklacing M-A formed along the grain boundaries in the ICHAZ of the ULCW joint (Figure 7) increases with increasing cooling rate, thereby leading to the lowest toughness [21]. Banded structures formed in the HAZ of the ULCW joint (Figure 4a) have a negative effect on the toughness [22]. Nevertheless, a low absorbed energy value (26.7 J) is also obtained for the ULCW joint with a notch in the FZ. The relatively high fraction of PF, and FSP (due to water quenching, see Figure 5e) in the FZ of the ULCW joint results in a decreasing toughness.

The absorbed energy values of the UDW specimens with a notch opened at the FZ and HAZ are higher than those of the ULCW joint with a notch opened at the FZ and HAZ at −40 °C. Compared with the HAZ of the ULCW joint, the HAZ of the UDW joint experiences a lower cooling rate (due to preheat, high inter-pass temperature, and air cooling). Therefore, the HAZ of the UDW joint contains a lower volume fraction of M and M-A constituents than the HAZ of the ULCW joint. Banded structures are absent from the HAZ of the UDW joint (as shown in Figure 4) and, hence, the toughness of this region is extremely high. In addition, the microstructure comprising the FZ of the UDW joint consists mainly of fine AF (see Figure 5a), which results in high toughness. This AF-dominated microstructure is more effective in preventing crack propagation [23].

**Figure 13:**
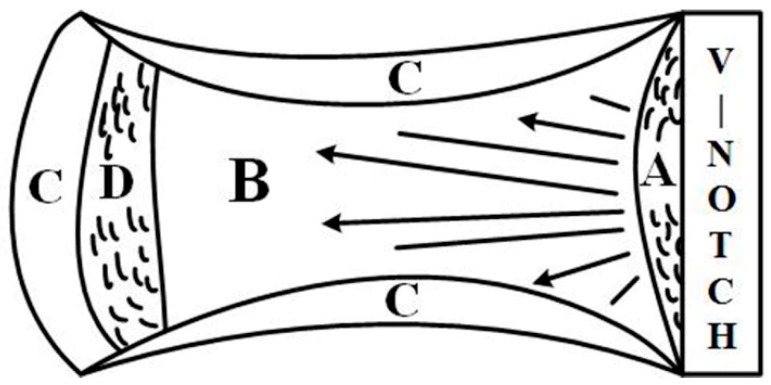
The screen zones of fracture surface after impact tests: fibrous zone (A), radial zone (B), shear lip zone (C) and second fibrous zone (D).

**Figure 14:**
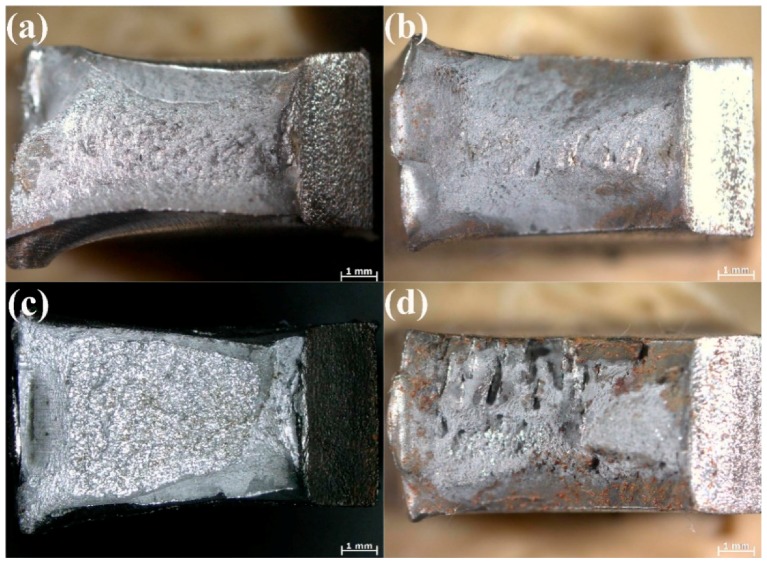
Impact macroscopic fracture morphology of welded joints with two welding methods: (**a**) HAZ-UDW joint; (**b**) FZ-UDW joint; (**c**) HAZ-ULCW joint; (**d**) FZ-ULCW joint.

The fracture surface after Charpy impact testing can be divided into four regions, i.e., the fibrous zone (A), radial zone (B), shear lip zone (C), and second fibrous zone (D), as shown in Figure 13. Generally, the fibrous zone and shear lip zone are expected to absorb higher impact energy than the radial zone [24]. The macro-fracture surfaces of the UDW and ULCW specimens subjected to impact testing at −40 °C are examined by optical microscopy, as shown in Figure 14. The fracture surface of the UDW specimen with a notch in the HAZ and the FZ is characterized by a large ductile fracture region and a small brittle fracture region. Compared with the corresponding zones of the ULCW specimen, the fibrous zone and shear lip zone constitute a larger fraction of the UDW specimen with a notch in the HAZ and the FZ, respectively. Therefore, the toughness values of the HAZ and FZ of the UDW joint are higher than those of the HAZ and FZ, respectively, of the ULCW joint. The brittle fracture region constitutes a very large fraction (~75–85%, see Figure 14c) of the ULCW specimen with a notch in the HAZ fracture surfaces. The fibrous zone and shear lip zone constitute small fractions. Many large cleavage cracks form in an edge (without a shear lip zone) of the ULCW specimen with a notch in the FZ, indicative of the poor toughness of this sample. The radial zone of the ULCW specimen with a notch in the FZ consists of a large brittle fracture region and a small ductile fracture region (Figure 14d). The fractographic observations submit a powerful substantiation for the Charpy impact testing results.

**Figure 15:**
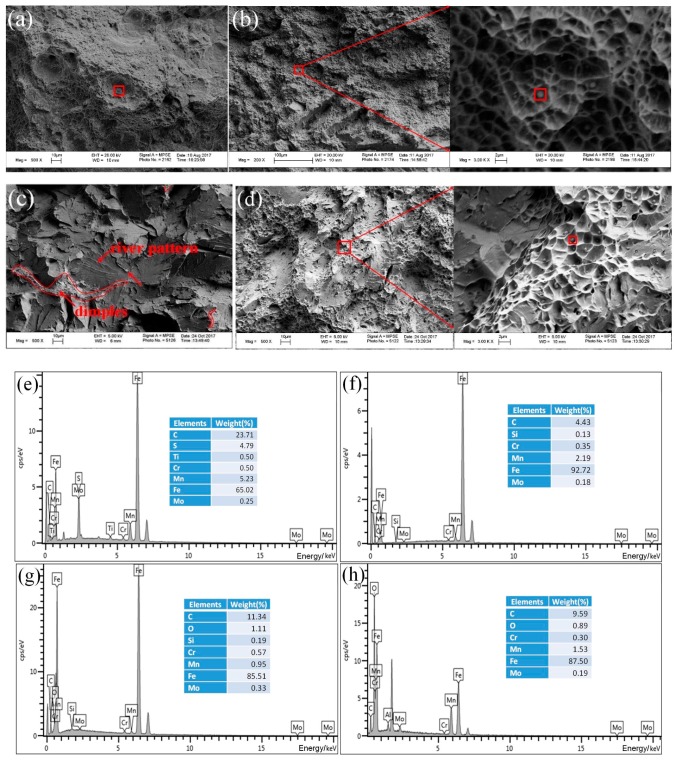
Fractured surfaces for the specimens after Charpy impact testing at −40 °C and the results of EDS analysis: (**a**) UDW specimen with notch at the HAZ; (**b**) UDW specimen with notch at the FZ; (**c**) ULCW specimen with notch at the HAZ; (**d**) ULCW specimen with notch at the FZ; (**e**) EDS spectrum for an inclusion particle in (**a**); (**f**) EDS spectrum for an inclusion particle in (**b**); (**g**) EDS spectrum for an inclusion particle in (**c**); (**h**) EDS spectrum for an inclusion particle in (**d**).

The Charpy impact fracture surfaces of the radial zone tested at −40 °C and the inclusion particles located in the microvoids are examined by SEM and EDS, respectively. High-magnification SEM images (Figure 15) are obtained of the final fracture zones of the HAZ and FZ of the UDW joint, as well as the FZ of the ULCW joint. As the figure shows, these regions are characterized by cleavage facets, ductile dimples, and microvoids, consistent with the occurrence of quasi-cleavage. The final fracture zone of the HAZ of the ULCW joint consists mainly of cleavage facets and a few dimples (Figure 15c), consistent with the occurrence of cleavage rupture. This indicates that the specimen fractured at relatively low values of the absorbed energy. The M-A constituents in the HAZ of the ULCW joint regions (see, for example, the smooth blocky particle indicated by the red arrow in Figure 15c) can act as the initiation sites of cleavage cracking. Compared with those formed in the HAZ of the ULCW joint, (i) a larger number of ductile dimples and microvoids are formed on the fracture surface of the HAZ of the UDW joint (see Figure 15a) and (ii) small equiaxed dimples and more cleavage facets formed in ULCW specimens with a notch opened at the FZ (see Figure 15d). The fracture morphology of the FZ regions of the UDW and the ULCW joints, as shown in Figure 15b or Figure 15d, reveals a mixed mode of cleavage facets and shear dimples. Compared with that of the FZ of the UDW-joint fracture zone, cleavage constitutes a larger area of the FZ in the ULCW joint-fracture zone. Therefore, the FZ of the UDW specimen has a higher impact toughness than the FZ of the ULCW specimen. 

Furthermore, EDS results indicate that the main elements composing the inclusion particles in the (i) HAZ of the UDW joint, (ii) FZ of the UDW joint, (iii) HAZ of the ULCW joint, and (iv) FZ of the ULCW joint are (i) C, S, Ti, Cr, Mn, Mo, Fe (Figure 15e), (ii) C, Si, Cr, Mn, Mo, Fe (Figure 15f), (iii) C, O, Si, Cr, Mn, Mo, Fe (Figure 15g), and (iv) C, O, Cr, Mn, Mo, Fe (Figure 15h).

## 4. Conclusions

In this study, workpieces of Q690E HSLA steel are welded by UDW and ULCW, respectively. The microstructure, micro-hardness, tensile strength, and impact toughness of the welded joints are determined. Based on the results, the following conclusions are reached:

Owing to the similar welding processes, the macrostructure of the ULCW joint is similar to that of the UDW joint. The microstructures of the FZ of the UDW joint and the ULCW joint consist mainly of three types of ferrite namely, AF, PF, and FSP. Compared with those of the UDW joint, the No. 4 and 5 passes of FZ within the ULCW joint yield more PF and FSP. Moreover, the prior austenite grain size of both types of joints decreases gradually with movement from the CGHAZ to the ICHAZ, and the microstructure in the HAZ consists mainly of B, M and TM.

The FZ, HAZ, and BM of both types of joints can be written in descending order of the average micro-hardness values, as follows: HAZ > FZ >BM. Furthermore, the micro-hardness values of the HAZ and FZ of the ULCW joint are higher than those of the HAZ and FZ of the UDW joint. The peak micro-hardness of the HAZ of the (i) ULCW joint occurs in the ICHAZ (peak value: ~430 HV_0.3_) and (ii) UDW joint occurs in the CGHAZ (peak value: ~360 HV_0.3_). In addition, the soft HAZ of the ULCW specimen is wider than the softened zone of the UDW specimen (~1.5 mm vs. ~1 mm).

The tensile strength of the UDW specimen is higher than the strengths of the ULCW joint. The UDW and ULCW specimens all failed in the soft HAZ. The wide softened zone (with relatively low micro-hardness) results in the low tensile strength of the ULCW specimen. The YS and UTS of the ULCW specimen are 109 MPa lower and 77 MPa lower, respectively, than those of the UDW joint.

The impact energy of the UDW joint is superior to that of the ULCW joint. The FZ and HAZ of the ULCW joint are brittle, owing to the generation of M and M-A in these regions.

These conclusions have some guiding significance for underwater local dry welding and underwater dry welding of Q690E. During the underwater welding production of Q690E, we could try to decrease the cooling rate of ULCW, which will decrease the fraction of PF and FSP in the FZ of the ULCW joint and improve the toughness of FZ. Post weld heat treatment could be used to improve the properties of the soft HAZs of Q690E underwater local dry welding joints.

## Figures and Tables

**Table 1:** Mechanical properties of Q690E steel.

Yield Stress (MPa)	Ultimate Tensile Strength (MPa)	Elongation (%)	Impact Toughness at −40 °C with Charpy V Specimen Dimensions of 55 mm × 10 mm × 5 mm (J)
750	819	16	83.5

**Table 2:** Chemical compositions of the base metal and welding filler wire (wt. %).

	C	Si	Mn	P	S	Cr	Ni	Al	Ti	Nb	Mo	V	Cu	Fe
Q690E steel	0.14	0.19	1.03	0.009	0.004	0.57	0.45	0.048	0.015	0.034	0.26	-	-	Bal.
Mn3Ni1CrMo	0.06	0.6	1.6	0.01	0.01	0.3	1.4	-	-	-	0.25	0.07	0.07	Bal.

**Table 3:** The welding parameters and the heat inputs.

Specimen No.	Welding Pass	Preheat Temperature before Welding (°C)	Inter-Pass Temperature (°C)	Arc Voltage (V)	Welding Current (A)	Welding Speed (mm s^−1^)	Heat Input (J mm^−1^)	The Depth of Water (m)
UDW joint	No. 1	100	100	21.8	180	5.6	595.6	0.3
	No. 2–5	/	100	23	200	5.6	698.2	0.3
ULCW joint	No. 1	/	/	21.8	180	5.6	595.6	0.3
	No. 2–5	/	/	23	200	5.6	698.2	0.3

**Table 4:** Tensile properties for the base material, the UDW joint and the ULCW joint.

Test Specimens	Yield Strength (MPa)	Tensile Strength (MPa)
Base material	843	896
UDW joint	831	876
ULCW joint	722	799

**Table 5:** Summary of absorbed energy results for the specimens at −40 °C.

Test Specimens	Average Absorbed Energy (J)	Variance
BM	83.5	3.04
UDW joint (Notch in the HAZ)	81	4.58
ULCW joint (Notch in the HAZ)	59.3	8.33
UDW joint (Notch in the FZ)	39	3
ULCW joint (Notch in the FZ)	26.7	1.16
